# PW03-009 – Genetics of PFAPA syndrome

**DOI:** 10.1186/1546-0096-11-S1-A235

**Published:** 2013-11-08

**Authors:** L Broderick, D Carvalho, A Magit, W Jiang, S Leuin, M Bothwell, D Kearns, S Pransky, HM Hoffman

**Affiliations:** 1 Medicine, University of California, San Diego, USA; 2 Surgery, University of California, San Diego, USA; 3 Ludwig Institute of Cancer Research, San Diego, USA; 4 Pediatrics, University of California, San Diego, San Diego, USA

## Introduction

Periodic Fever, Aphthous stomatitis, Pharyngitis and Adenitis (PFAPA) syndrome is an autoinflammatory disorder of childhood and little is known about the underlying etiology. While mutations involving the IL-1 pathway have been identified in other recurrent fever disorders, including TNF-receptor associated periodic syndrome (TRAPS) and cryopyrin-associated periodic syndrome (CAPS), PFAPA syndrome is not traditionally considered to be a hereditary fever disorder.

## Objectives

To evaluate pediatric patients with PFAPA for family histories suggestive of immune dysregulation and to correlate inheritability with immune phenotype.

## Methods

Patient data and detailed family histories were collected for over 170 children with recurrent fevers including 70 patients with PFAPA to create a prospective cohort over a 4-year period. DNA was isolated from blood or tonsillar tissue from recurrent fever patients, and *NLRP3* and *TNFRSF1A* were sequenced. Quantitative real time PCR was used to evaluate *IL-36* transcripts in tonsils.

## Results

Our cohort reflects the diversity of San Diego, without predilection for any specific ethnic background. Family histories revealed 21% of patients have a first degree relative with recurrent fevers and 12% with tonsillitis in childhood, with only 1.4% reporting a history of recurrent infections. We have identified over 30 families with 2-8 affected members. These patients do not possess mutations commonly seen in other autoinflammatory disorders such as CAPS or TRAPS, suggesting that a novel gene may be involved. Upregulation of IL-36 mRNA expression in tonsils identifies the IL-1 family member IL-36 as a candidate gene.

## Conclusion

A substantial portion of our families with PFAPA report childhood histories of recurrent fevers that resolved either spontaneously or with tonsillectomy, indicating a possible dominantly inherited trait that impacts the developing immune system, including the tonsils.

## Disclosure of interest

L. Broderick: None declared, D. Carvalho: None declared, A. Magit: None declared, W. Jiang: None declared, S. Leuin: None declared, M. Bothwell: None declared, D. Kearns: None declared, S. Pransky: None declared, H. Hoffman Consultant for: Regeneron, Novartis, and Sobi

